# Nesprin 1α2 is essential for mouse postnatal viability and nuclear positioning in skeletal muscle

**DOI:** 10.1083/jcb.201612128

**Published:** 2017-07-03

**Authors:** Matthew J. Stroud, Wei Feng, Jianlin Zhang, Jennifer Veevers, Xi Fang, Larry Gerace, Ju Chen

**Affiliations:** 1 Department of Medicine, University of California, San Diego, La Jolla, CA; 2 Department of Cell and Molecular Biology, The Scripps Research Institute, La Jolla, CA

## Abstract

The position of the nucleus in a cell is controlled by interactions between the linker of nucleoskeleton and cytoskeleton (LINC) complex and the cytoskeleton. Defects in nuclear positioning and abnormal aggregation of nuclei occur in many muscle diseases and correlate with muscle dysfunction. Nesprin 1, which includes multiple isoforms, is an integral component of the LINC complex, critical for nuclear positioning and anchorage in skeletal muscle, and is thought to provide an essential link between nuclei and actin. However, previous studies have yet to identify which isoform is responsible. To elucidate this, we generated a series of nesprin 1 mutant mice. We showed that the actin-binding domains of nesprin 1 were dispensable, whereas nesprin 1α2, which lacks actin-binding domains, was crucial for postnatal viability, nuclear positioning, and skeletal muscle function. Furthermore, we revealed that kinesin 1 was displaced in fibers of nesprin 1α2–knockout mice, suggesting that this interaction may play an important role in positioning of myonuclei and functional skeletal muscle.

## Abstract (precis)

Defects in nuclear positioning occur in muscle diseases and correlate with muscle dysfunction. In this study, Stroud et al. show that nesprin 1α2 is the fundamental nesprin 1 isoform for nuclear positioning, skeletal muscle function, and postnatal viability.

## Introduction

The position of the nucleus in a cell is controlled by interactions between the nuclear envelope (NE) and the cytoskeleton (Crisp et al., 2006; Starr and Fridolfsson, 2010; Gundersen and Worman, 2013; Stroud et al., 2014a; Wilson and Holzbaur, 2015). Aberrant nuclear positioning is frequently associated with cell dysfunction and can have clinical consequences (Cohn and Campbell, 2000; Romero, 2010; Gundersen and Worman, 2013). Several muscle diseases are correlated with aberrant nuclear positioning (Cohn and Campbell, 2000; Shah et al., 2004; Zhang et al., 2007b, 2010; Puckelwartz et al., 2009; Romero, 2010; Mattioli et al., 2011; Metzger et al., 2012; Gundersen and Worman, 2013), suggesting that proper nuclear localization and anchorage is essential for normal skeletal muscle function. As myoblast fusion occurs to give rise to muscle fibers, microtubules mediate the movement of nuclei through the cell to become anchored under the sarcolemma at the cell periphery in mature muscle fibers (Englander and Rubin, 1987; Reinsch and Gönczy, 1998; Morris, 2003; Starr, 2009; Wilson and Holzbaur, 2012). Individual nuclei are arrayed within a mature muscle fiber so as to maximize the internuclear distance, perhaps to facilitate even dispersion of molecules from nuclei to cytoplasm (Bruusgaard et al., 2003).

**Figure 1.**
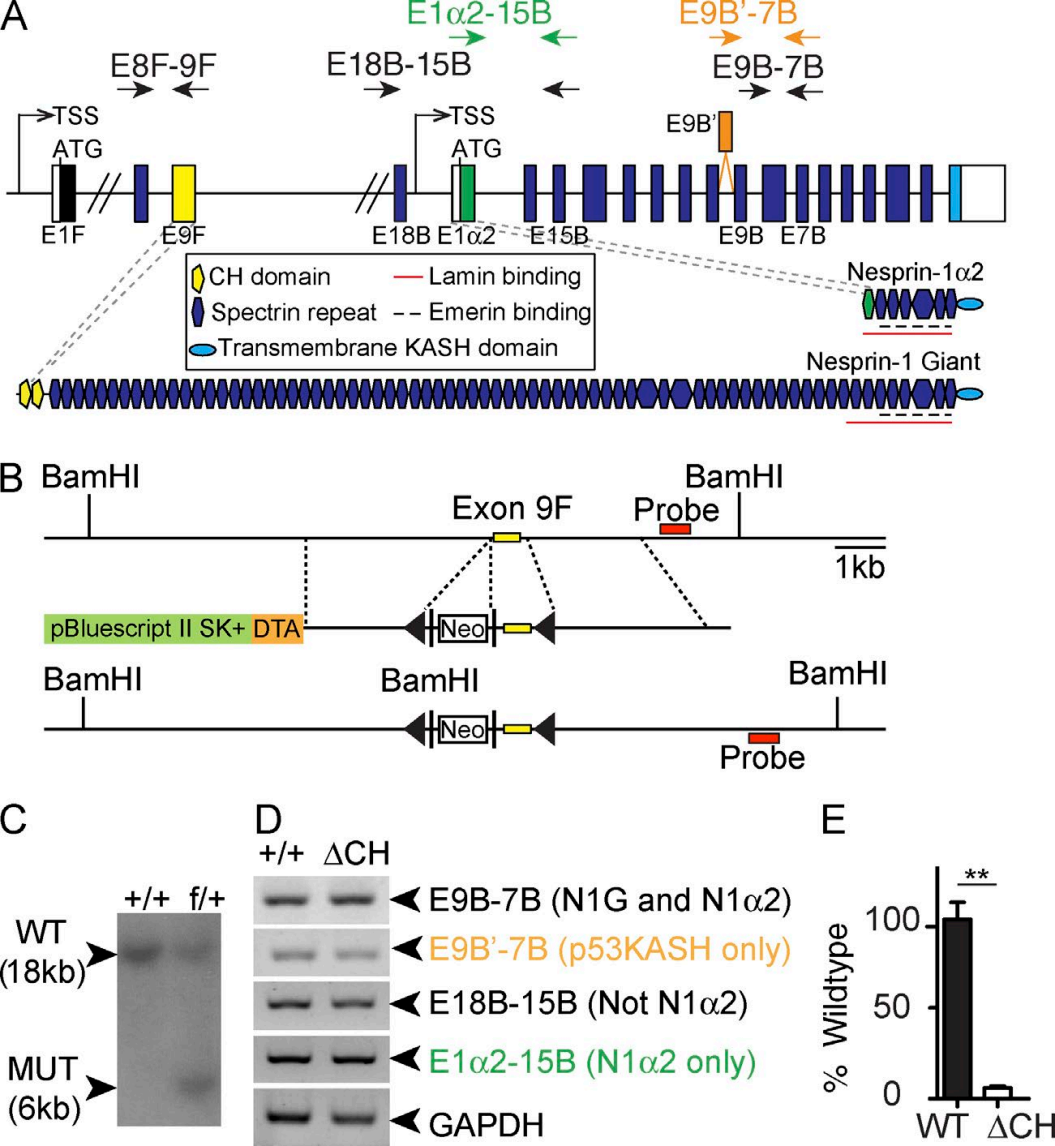
**Generation of mice lacking nesprin 1 CH domains.** (A) Schematic of syne1 gene with primer locations used for PCRs in D and E (arrows). (B) Construct used for targeting the *syne1* gene, with the exon 9F (yellow rectangle) flanked by two LoxP sites (arrowheads). DTA, diphtheria toxin A; black rectangles, flippase recombination target sites; Neo, neomycin cassette. (C) Southern blot confirmation of the WT allele at 18 kb and presence of the mutant allele (MUT) at 6 kb. (D) Semiquantitative RT-PCR of WT and nesprin 1ΔCH mRNA isolated from skeletal muscle. Note that similarly to WT and as expected, the other nesprin 1 isoforms were present in nesprin 1ΔCH mRNA. (E) qRT-PCR of mRNA from WT and nesprin 1ΔCH using primers specific to the CH domain–encoding exon 9F. Note the significantly decreased levels of nesprin 1ΔCH domain–containing exon 9F compared with WT. **, P < 0.01 according to an unpaired Student’s t test.

NE spectrin repeat (SR) proteins, or nesprins, are a family of four NE proteins that are integral components of the linker of nucleoskeleton and cytoskeleton (LINC) complex (Zhang et al., 2001, 2005, 2010; Rajgor et al., 2012). Alternative transcription initiation, termination, and RNA splicing of the *syne-1* gene (encoding for nesprin 1) generate multiple isoforms that vary greatly in size (Warren et al., 2005; Simpson and Roberts, 2008; Rajgor et al., 2012). The largest, or giant (G), isoform of nesprin 1 (nesprin 1G) consists of an N-terminally paired actin-binding calponin homology (CH) domain, a central SR-containing rod domain, and a C-terminal transmembrane Klarsicht, ANC-1, and Syne homology (KASH) domain that interacts with Sad1/UNC-84 (SUN) domain proteins, which bind to nuclear lamins (Padmakumar et al., 2004; Sosa et al., 2012). Other nesprin 1 isoforms that lack either the N-terminal CH domains, the C-terminal KASH domain, or both vary markedly in the length of the SR-containing rod domain (Warren et al., 2005; Simpson and Roberts, 2008; Rajgor et al., 2012). Nesprin 1G and nesprin 1α2 are the predominant isoforms of nesprin 1 expressed in skeletal muscle (Padmakumar et al., 2004; Randles et al., 2010; Duong et al., 2014). Nesprin 1α2 (also named syne-1A [Apel et al., 2000] or myne-1 [Mislow et al., 2002]) is an understudied short isoform that contains seven SRs and the KASH domain but lacks the actin-binding CH domains (Fig. 1 A; Apel et al., 2000; Mislow et al., 2002; Zhang et al., 2007a; Rajgor et al., 2012).

We and others have previously shown that nesprin 1 is critical for nuclear positioning and anchorage in skeletal muscle (Zhang et al., 2007b, 2010; Puckelwartz et al., 2009). Notably, loss of all known nesprin 1 isoforms led to postnatal lethality in 60% of newborn pups, and surviving mice developed skeletal myopathy (Zhang et al., 2010). Nesprins are thought to regulate nuclear anchorage by providing a critical link between nuclei and the actin cytoskeleton (Zhang et al., 2002, 2007b, 2010; Zhen et al., 2002; Padmakumar et al., 2004; Puckelwartz et al., 2009; Banerjee et al., 2014); therefore, previous approaches to study the role of nesprin 1 in skeletal muscle either interfered with the KASH domain (Zhang et al., 2007b; Puckelwartz et al., 2009) or ablated all nesprin 1 isoforms (Zhang et al., 2010). However, there is currently no direct evidence to suggest nesprin 1G links the nucleoskeleton to actin filaments in skeletal muscle, and current studies preclude the understanding as to which isoform of nesprin 1 is critical for skeletal muscle function.

To address this question, and to investigate the in vivo function of different nesprin 1 isoforms, we generated nesprin 1ΔCH domain–specific knockout (KO; nesprin 1ΔCH^−/−^) mice in which the exon encoding the actin-binding CH domains of nesprin 1 was ablated as well as nesprin 1α2 isoform-specific deficient mice. We show that the CH domains of nesprin 1 are dispensable for postnatal viability, nuclear positioning, and skeletal muscle function. In contrast, loss of nesprin 1α2 led to severe nuclear mispositioning and postnatal lethality. Interestingly, we found that the microtubule motor protein kinesin 1 is specifically mislocalized in nesprin 1α2^−/−^ muscle fibers but remains at the NE in skeletal muscles of nesprin 1ΔCH^−/−^ mice. These data suggest that nesprin 1α2 plays a fundamental role in vivo and is the critical nesprin 1 isoform essential for skeletal muscle function. Furthermore, Nesprin 1α2 interacts with kinesin 1 to facilitate the nuclear dynamics necessary to position nuclei for normal skeletal muscle function.

## Results and discussion

### The CH domains of nesprin 1 are dispensable for perinatal viability and nesprin 1 function

The N-terminal tandem CH domains of nesprin 1G have been shown to interact with actin in vitro (Padmakumar et al., 2004). We therefore hypothesized that similar to our nesprin 1 global KO (GKO) model (Zhang et al., 2010), nesprin 1ΔCH^−/−^, lacking the ability to bind actin through their CH domains of nesprin 1G, would exhibit perinatal lethality.

To test this hypothesis, we floxed exon 9F, which encodes for the latter CH domain in nesprin 1, with two LoxP sites and crossed it with *Sox2*-Cre deleter mice to globally ablate expression of CH domain–containing isoforms (Fig. 1, B and C). Owing to our inability to detect full-length nesprin 1G (1.2 MD) proteins in mouse skeletal muscle, and to ensure that the CH domains were appropriately ablated in nesprin 1ΔCH^−/−^ mutants, we performed detailed RT-PCR analysis. Importantly, we detected no changes in expression levels of other nesprin 1 isoforms after removal of exon 9F (Fig. 1 D), and expression of the CH domain–containing nesprin 1 isoform was abolished (Fig. 1 E). Surprisingly, and in contrast to our previous nesprin 1 KO model, nesprin 1ΔCH^−/−^ mice were born at normal Mendelian ratios, displayed no perinatal lethality nor loss in bodyweight compared with their wild-type (WT) littermates, and survived up to 18 mo of age with no overt skeletal or cardiac muscle dysfunction (not depicted).

These data demonstrate that the CH domains of nesprin 1 are dispensable for viability and indicate that the lack of actin-binding ability of nesprin 1G is not sufficient to explain the perinatal lethality and skeletal muscle weakness observed in nesprin 1 KO mice (Puckelwartz et al., 2009; Zhang et al., 2010).

### LINC complex protein levels, localization, and nuclear shape and positioning are unaffected in nesprin 1ΔCH^−/−^ mice

**Figure 2.**
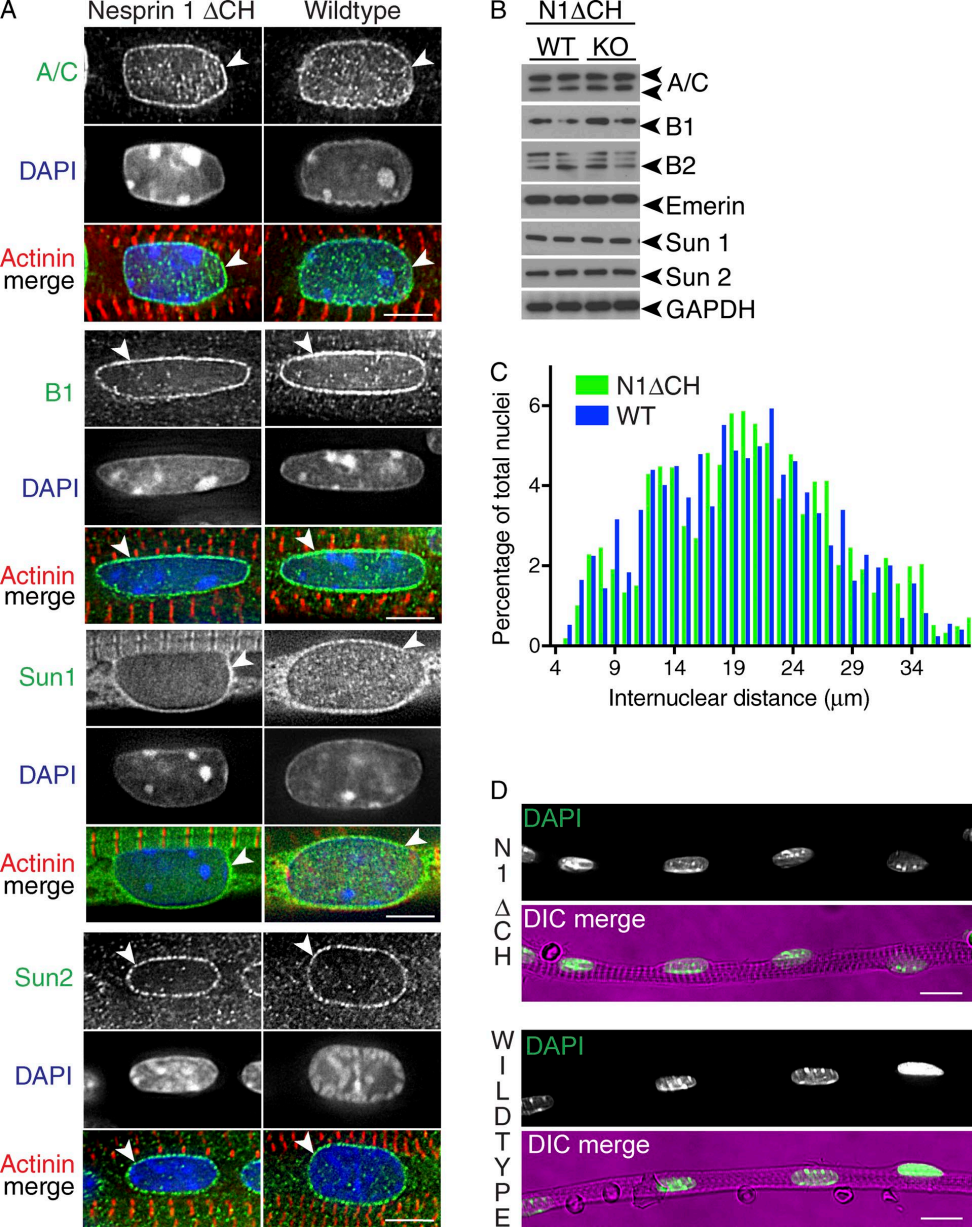
**Nuclear shape, internuclear distances, and LINC complex proteins were unaffected in TA muscles from nesprin-1ΔCH mice.** (A) TA skeletal muscle fibers isolated from either WT or nesprin 1ΔCH (N1ΔCH) mice were fixed and stained using Lamin A/C (A/C), Lamin B1 (B1), Sun1, Sun2 (green), and α-actinin (red) antibodies. Arrowheads denote the specific localization to the NE. (B) Western blots of the LINC complex proteins Sun1 and Sun2, the LINC-associated protein emerin, and the nuclear lamins A/C, B1, and B2 from WT and nesprin 1ΔCH (KO) TA muscle lysates. Note that the localization and levels were unaffected in nesprin 1ΔCH compared with WT controls. GAPDH served as a loading control. (C) Internuclear distances were quantified in TA muscle fibers isolated from WT (blue bars) and nesprin 1ΔCH (green bars) mice shown in A. Note that the distances between nuclei were unaffected. *n* = 176–205 and *n* = 140–230 internuclear distances were counted for nesprin 1ΔCH and WT, respectively. (D) Low-magnification representative images of those shown in A. *n* = at least three to four pups per genotype for each panel. Bars: (A) 5 µm; (D) 10 µm. DIC, differential interference contrast.

Because our data indicated that the CH domains of nesprin 1 are not responsible for the skeletal muscle phenotype observed in nesprin 1 GKO mice, we next investigated the effects of loss of CH domain–containing nesprin 1 isoforms on LINC complex protein expression levels and subcellular localization in skeletal muscle fibers. As shown in Fig. 2 A, the localization of the LINC complex proteins SUN1 and SUN2 and nuclear lamins A/C and B1 were unchanged in nesprin 1ΔCH^−/−^ tibialis anterior (TA) muscle fibers at embryonic day 18.5 (E18.5). Complementary to our immunofluorescence data, Western blotting and quantitative RT-PCR (qRT-PCR) analysis confirmed that expression of LINC complex components was unchanged in nesprin 1ΔCH^−/−^ mice (Figs. 2 B and S1). Furthermore, there was no significant difference in internuclear distance, as calculated by measuring the distance from the center of one nucleus to that of neighboring nuclei, in nesprin 1ΔCH^−/−^ TA muscle fibers (mean distance of 21.4 µm) when compared with WT littermates (mean distance of 20.3 µm; Fig. 2, C and D). Quantification also revealed that individual nuclei in mutant (nuclear length 7.9 µm) and control (nuclear length 7.7 µm) skeletal muscles were of similar size and shape.

These data demonstrate that the CH domains of nesprin 1 are dispensable for nuclear shape and positioning as well as localization and levels of LINC complex and associated proteins. Thus, nesprin 1G and other CH domain–containing nesprin 1 isoforms are not required for normal skeletal muscle structure and function. This is interesting because others have reported that nesprin 1G is expressed and localizes to the Z disc in skeletal muscle (Zhang et al., 2002; Padmakumar et al., 2004). Importantly, our data suggest that another nesprin 1 isoform or isoforms, such as nesprin 1α2, and/or a cytoskeleton protein or proteins other than actin play critical roles for normal skeletal muscle function. Nesprin 1G may play a tissue-specific role in the brain, where it was recently reported to be expressed in the cerebellum (Razafsky and Hodzic, 2015). In light of this, it would be interesting to investigate whether nesprin 1ΔCH^−/−^ mice develop neurological abnormalities.

### Nesprin 1α2 is essential for perinatal viability

**Figure 3.**
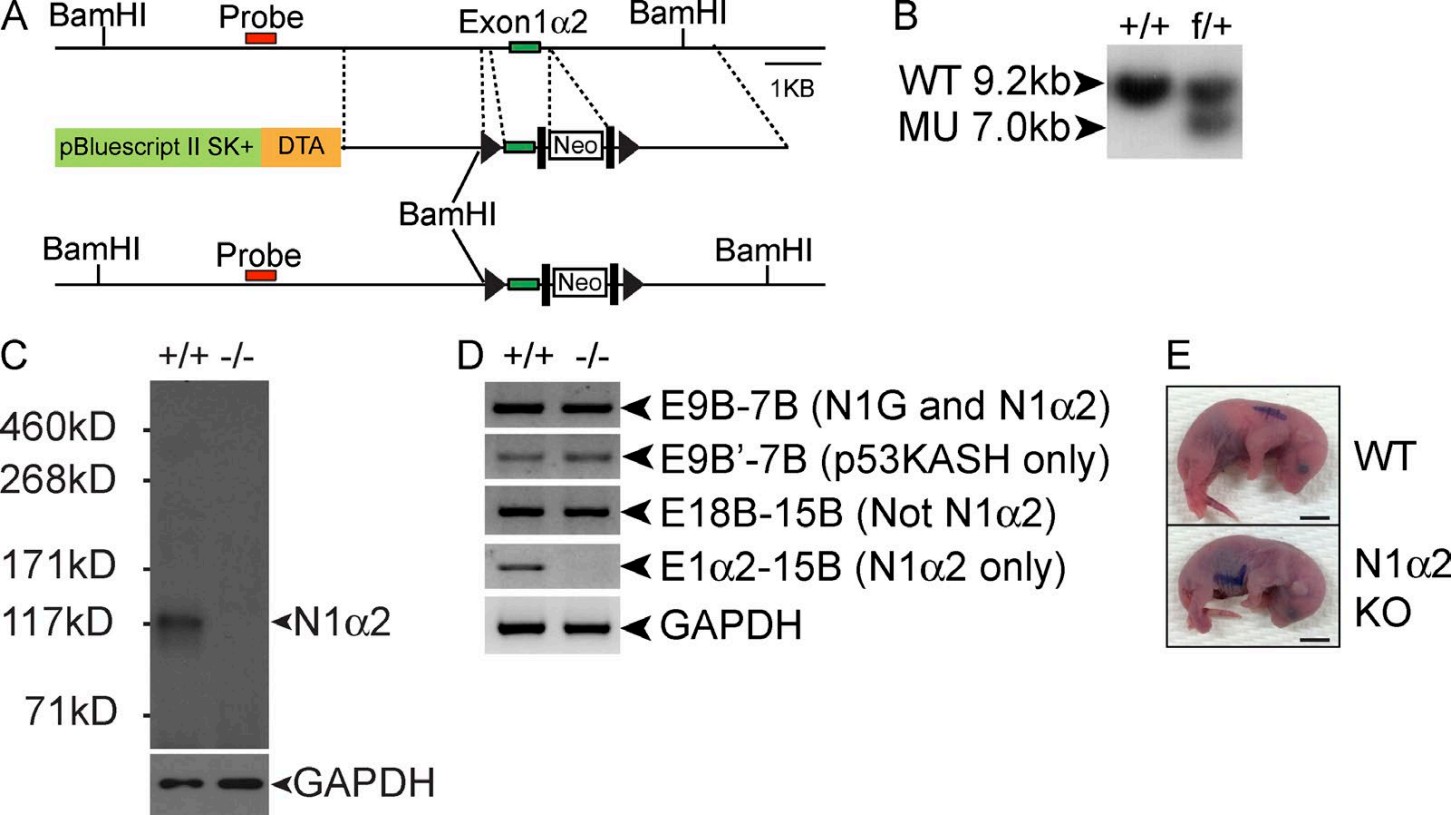
**Nesprin 1α2 KO mice died perinatally.** (A) Construct used for targeting the *nesprin 1* gene, with exon 1α2 flanked by two LoxP sites (arrowheads). DTA, diphtheria toxin A; black rectangles, flippase recombination target sites; Neo, neomycin cassette. (B) Southern blot confirmation of the WT allele at 9.2 kb and the presence of the mutant allele (MU) at the predicted size of 7 kb. (C) Western blot of skeletal muscle lysates from WT (+/+) and nesprin 1α2 KO (−/−) mice using a nesprin 1 antibody. An arrowhead indicates the predicted molecular weight of nesprin 1α2. Note the absence of the band at the predicted molecular weight of nesprin 1α2. GAPDH served as a loading control. (D) Semiquantitative RT-PCR of WT and nesprin 1α2 KO mRNA isolated from skeletal muscle. Note that similarly to WT and as expected, other nesprin 1 isoforms were present in nesprin 1α2 KO mice; however, the nesprin 1α2 isoform was absent. (E) E18.5 embryos were extracted from pregnant females and imaged after 5 min. Note that WT embryos were able to breathe and turned pink shortly after delivery, whereas nesprin 1α2 KO embryos turned cyanotic (blue). Bars, 5 mm.

To investigate the in vivo function of nesprin 1α2, we generated mice in which the first exon that is unique to nesprin 1α2 was flanked by two LoxP sites (Fig. 3, A and B). We crossed these mice with *Sox2* deleter mice to generate heterozygous null mutant nesprin 1α2 mice (nesprin 1α2^+/−^), which were then intercrossed to generate homozygous null mice (nesprin 1α2^−/−^). Western blot analysis demonstrated that nesprin 1α2 was absent in nesprin 1α2^−/−^ mice (Fig. 3 C). To ensure that other isoforms were unaffected after removal of the nesprin 1α2–specific exon, we performed RT-PCR on mRNA isolated from nesprin 1α2^−/−^ and WT littermates. As shown in Fig. 3 D, mRNA expression of other nesprin 1 isoforms was unchanged, whereas mRNA expression of the nesprin 1α2 isoform was undetectable and therefore deleted. Of 68 mice obtained from crossing nesprin 1α2^+/−^ heterozygotes, we would have expected 17 nesprin 1α2^−/−^ mutants; however, we only recovered two mutants at weaning (P21). We did observe the expected Mendelian ratios (10/41) at E18.5. Upon embryo extraction, we observed that WT embryos rapidly turned pink and started to breathe, whereas all of the nesprin 1α2^−/−^ embryos remained cyanotic (blue) despite stimulation and died within 5 min of cesarean section (Fig. 3 E). The two nesprin 1α2^−/−^ mice that survived after P21 were smaller, had reduced body weight, and developed kyphosis, indicating skeletal muscle dysfunction. No physiological analyses were performed on the surviving mice per se; however, given that the observed phenotype was similar to our previous nesprin 1 GKO mouse (Zhang et al., 2010), we postulate that surviving nesprin 1α2^−/−^ mice likely had dysfunctional skeletal muscle.

No cardiac dysfunction was observed in the nesprin 1α2^−/−^ mice, similar to our nesprin 1 GKO mice. Heart weight/body weight ratios in the nesprin 1α2^−/−^ embryos were normal, and histological analyses revealed no gross morphological defects in nesprin 1α2^−/−^ mice compared with control littermates (unpublished data). Furthermore, no overt cardiac defects were observed after ablation of nesprin 1α2 expression in cardiomyocytes up to 14 mo of age (unpublished data). These data are in agreement with our previous findings that simultaneous deletion of both nesprins 1 and 2 is required to affect cardiac function (Banerjee et al., 2014).

Our data suggest that loss of nesprin 1α2 phenocopies the perinatal lethality observed in nesprin 1 GKO mice (Zhang et al., 2010). Conversely, nesprin 1ΔCH^−/−^ mice do not display any overt phenotypes. As nesprin 2 expression has been reported in skeletal muscle (Lüke et al., 2008), we postulate that it may compensate for the loss of nesprin 1ΔCH. As we have not been able to reliably detect nesprin 2 in skeletal muscle with available antibodies, we performed RT-PCR, which revealed that the levels of short and long nesprin 2 isoforms were unchanged in nesprin 1ΔCH^−/−^ mice (Fig. S2), suggesting that compensation by up-regulation of nesprin 2 does not occur. mRNA levels of short and long nesprin 2 isoforms were also unchanged in the nesprin 1 GKO and the nesprin 1α2^−/−^ mice (Fig. S2).

The difference in survival rates at weaning between nesprin 1 GKO (40%) versus nesprin 1α2^−/−^ mice (12%) most likely results from the different mouse backgrounds of the two mouse lines. Nesprin 1 GKO mice were a mix of 129X1/SvJ and Black Swiss, whereas nesprin 1α2^−/−^ mice were in a mixed 129X1/SvJ and C57BL/6J background. Future studies will be required to pinpoint the exact cause of death of the nesprin 1α2^−/−^ mice using tissue-specific KOs of nesprin 1α2 in skeletal muscle and neurons, respectively.

### Nuclei are mispositioned in nesprin 1α2^−/−^ muscle, but the majority of LINC complex protein localization and levels are unchanged

**Figure 4.**
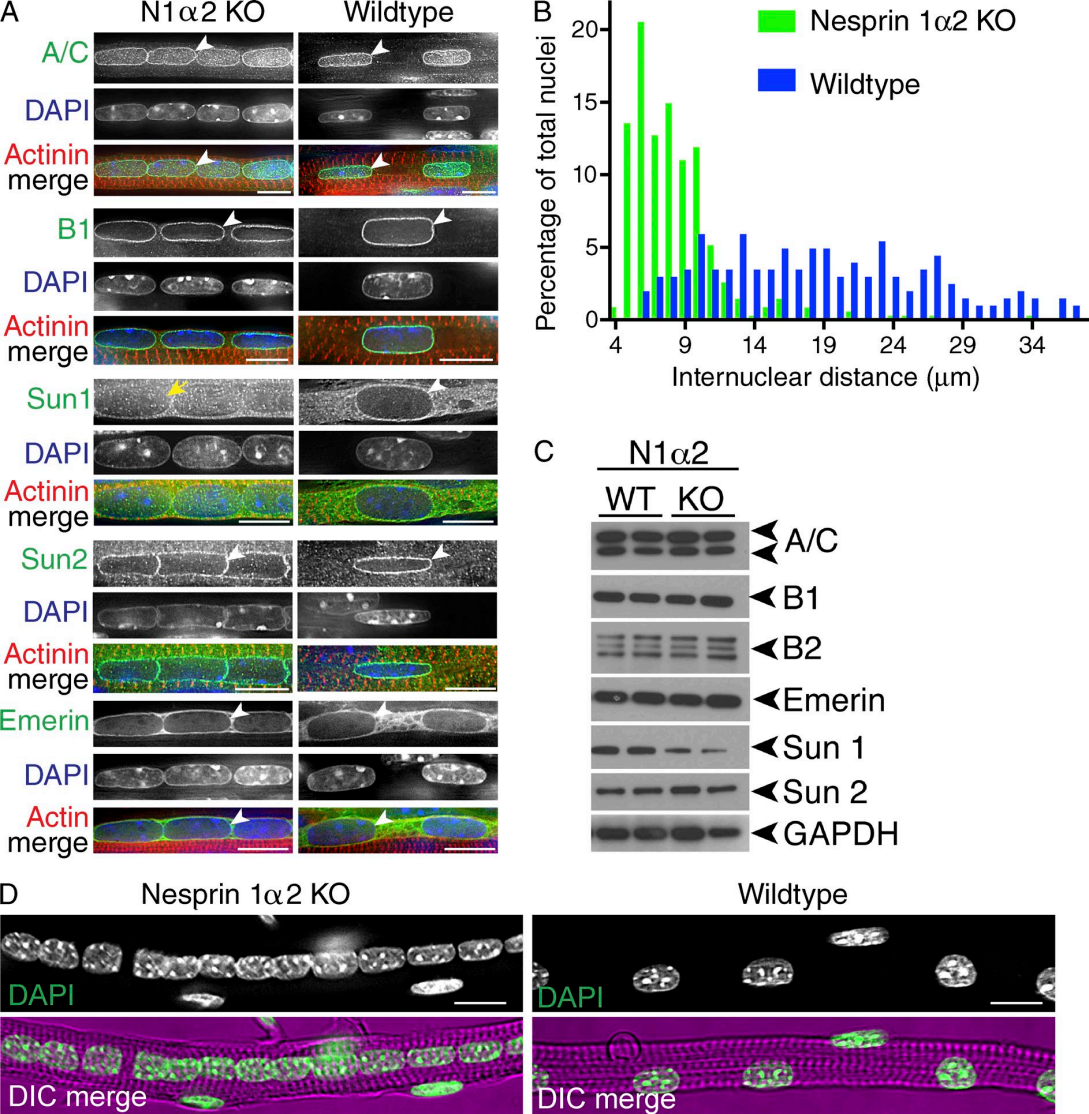
**Nuclear positioning was affected in TA muscles from nesprin 1α2 KO mice.** (A) TA skeletal muscle fibers isolated from either WT or nesprin 1α2 KO (N1α2 KO) mice were fixed and stained using Lamin A/C (A/C), Lamin B1 (B1), Sun1, Sun2, emerin (green), andαα-actinin (red) antibodies. Note the striking differences in nuclear positioning between WT and KO fibers but similar localization of the LINC-associated components (arrowheads). Sun1 localization was more cytoplasmic in KO TAs compared with controls (yellow arrows). (B) Quantification of internuclear distances of nesprin 1α2 KO (green bars) and WT mice (blue bars) shown in A. Note the striking clustering of nuclei in nesprin 1α2 KO myofibrils. *n* = 220–436 and *n* = 201–226 internuclear distances were counted for nesprin 1α2 KO and WT, respectively. (C) Western blots of the LINC complex proteins Sun1 and Sun2, the LINC-associated protein emerin, and the nuclear lamins A/C, B1, and B2 from WT and nesprin 1α2 KO TA muscle lysates. Note that the levels of most of the proteins were unaffected except Sun1, which was down-regulated. GAPDH served as a loading control. (D) Low-magnification representative images of those shown in A. *n* = at least three to four pups per genotype for each panel. Bars: (A) 5 µm; (D) 10 µm. DIC, differential interference contrast.

To determine the role of nesprin 1α2 in nuclear morphology and positioning as well as LINC complex protein expression and subcellular localization, we performed detailed analyses of isolated TA muscles at E18.5. The most striking defect we consistently observed in the nesprin 1α2^−/−^ mice was the clustering of nuclei in myofibers (Fig. 4, A–C; and Videos 1, 2, and 3). Nuclear lengths were unchanged (nesprin 1α2^−/−^, 7.8 µm; WT, 8.8 µm); however, the internuclear distance was significantly reduced from 20.5 µm in WT to 8.8 µm in nesprin 1α2^−/−^ mice. These data suggest that nesprin 1α2 plays a critical role in nuclear positioning akin to the global loss of nesprin 1 (Zhang et al., 2007b, 2010; Puckelwartz et al., 2009). To examine how the loss of nesprin 1α2 affected the LINC complex and nuclear lamina, we performed immunofluorescence, Western blotting, and qRT-PCR analyses on isolated TA muscles. We observed that in both nesprin 1α2^−/−^ and WT TA muscle, the localization and expression levels of the nuclear lamins A/C, B1, SUN2, and emerin remained associated with the NE (arrowheads in Fig. 4, A and D; also see Fig. S1). Conversely, SUN1 appeared more cytoplasmic in the nesprin 1α2^−/−^ muscles (Fig. 4 A, yellow arrows) and was expressed at lower levels (Fig. 4, A and D), suggesting that nesprin 1α2 may preferentially interact with SUN1 over SUN2 at the NE. Collectively, these data suggest that nesprin 1α2 is the critical nesprin 1 isoform for skeletal muscle function.

### Kinesin 1 is displaced from the NE in nesprin 1α2^−/−^ fibers, suggesting that it may govern positioning of myonuclei

The related family member nesprin 2 has been shown in cell lines to interact with the microtubule plus end–directed motor protein kinesin 1 via a four-residue tryptophan-acidic LEWD motif that is also present in nesprin 1α2 (Schneider et al., 2011; Wilson and Holzbaur, 2015). Interestingly, phenotypes of mice lacking kinesin 1 in skeletal muscle are strikingly similar to those we observed in nesprin 1α2^−/−^ mice, including abnormal nuclear aggregation and perinatal lethality (Wang et al., 2013).

**Figure 5.**
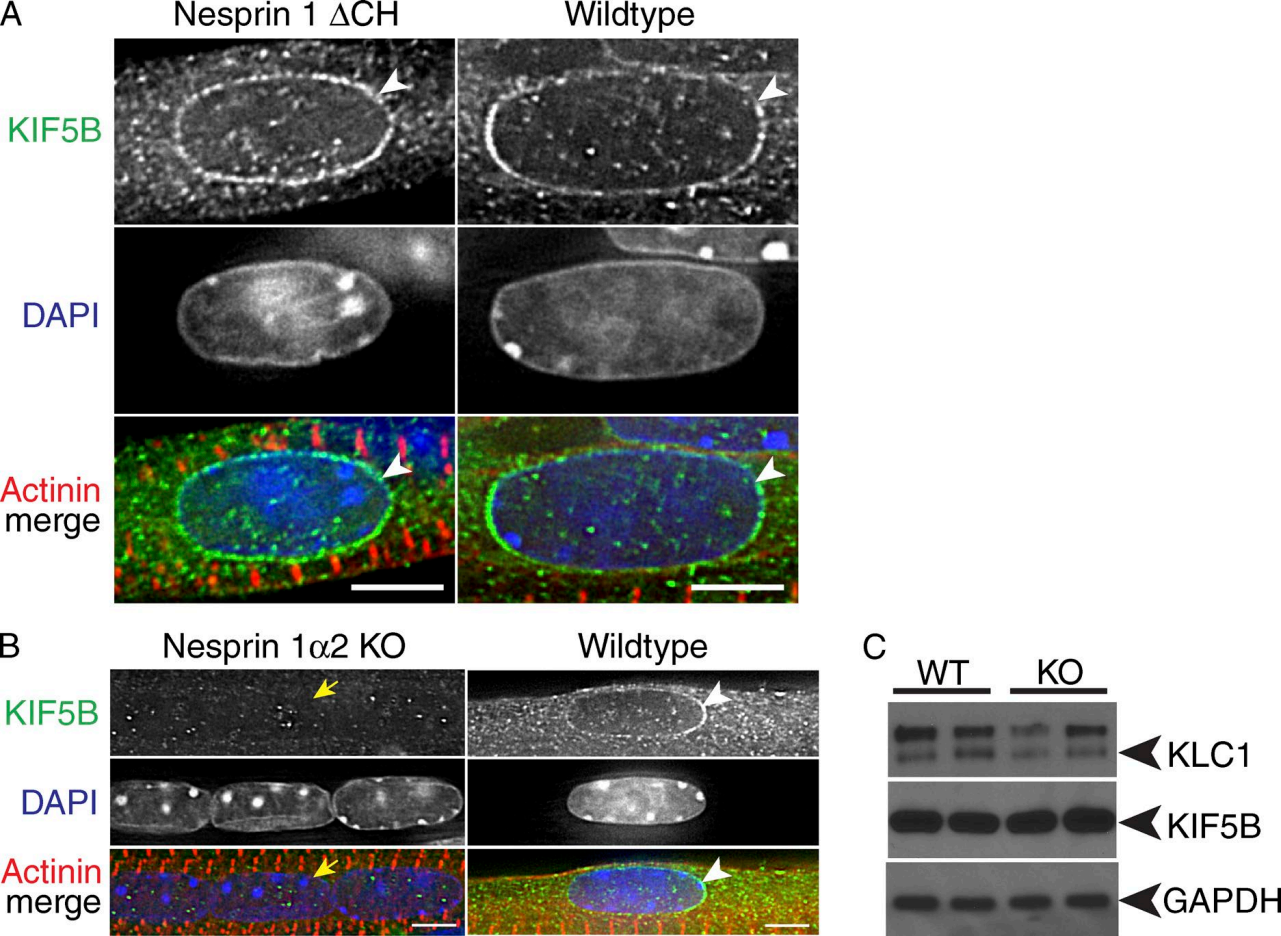
**Kinesin 1 localization was altered in TA muscles from nesprin 1α2 KO but not in WT or nesprin 1ΔCH^−/−^.** (A and B) TA skeletal muscle fibers isolated from WT, nesprin 1ΔCH (A), and nesprin 1α2 KO (B) mice were fixed and stained using an antibody directed to the heavy chain of kinesin 1 (KIF5B) and α-actinin (red). Note that kinesin 1 localized to the NE in both WT and nesprin 1ΔCH (arrowheads) but was diffuse in nesprin 1α2 KO TA muscles (yellow arrows). (C) Western blots of KLC1 and KIF5B from WT and nesprin 1α2 KO TA muscles. Note that the levels of KLC1 were slightly down-regulated, whereas KIF5B levels were unchanged. GAPDH served as a loading control. *n* = at least three to four pups per genotype for each panel. Bars, 5 µm.

To investigate whether loss of nesprin 1α2 would lead to changes of kinesin 1, we isolated TA muscle fibers from E18.5 embryos and stained them with an antibody specific to the kinesin 1 heavy chain KIF5B. As predicted, KIF5B localized to the NE in E18.5 TA fibers isolated from WT and nesprin 1ΔCH^−/−^ mice (Fig. 5 A, arrowheads). Conversely, KIF5B was completely lost from the NE in nesprin 1α2^−/−^ TA fibers (Fig. 5 B, yellow arrows). Interestingly, the levels of KIF5B and KLC1/2 were unchanged in TA fibers isolated from nesprin 1α2^−/−^ compared with WT (Fig. 5 C). Importantly, microtubule organization was not affected in the muscle fibers, and nesprin 2 levels of short and long isoforms were unchanged in nesprin 1ΔCH^−/−^ and nesprin 1α2^−/−^ mice, suggesting that it is unable to compensate (Figs. S2 and S3).

Collectively, our results indicate that nesprin 1α2 is critical for the perinuclear localization of kinesin 1, suggesting that interaction of nesprin 1α2 with kinesin 1 may play an essential role in myonuclear positioning and skeletal muscle function.

It is important to point out that, although we can detect mRNA for multiple different nesprin 1 isoforms in skeletal muscle, we can only detect the nesprin 1α2 isoform with a monoclonal antibody (MANNES1E 7A12; Fig. 3 C). In agreement with our results, Western blot data performed by others on C2C12 myotube extracts using the same monoclonal antibody and another rabbit anti–nesprin 1 polyclonal antibody clearly showed that only a 115-kD isoform of nesprin 1 corresponding with the size of nesprin 1α2 could be detected (Espigat-Georger et al., 2016). Although the monoclonal antibody was raised against full-length nesprin 1α2, specific epitopes recognized by the antibody have not been clearly mapped (Randles et al., 2010). The epitope or epitopes for the rabbit polyclonal antibody also remain unknown. Thus, there remains a possibility that other nesprin 1 isoforms that are not detectable by these antibodies are present in skeletal muscle. However, as we see clear mislocalization of kinesin 1 in our nesprin 1α2 mutant mice, we do not think that the putative nesprin isoforms can compensate for the interaction of nesprin 1α2 with kinesin.

In summary, we show that the CH domains of nesprin 1 are not required for proper nuclear localization or skeletal muscle function in vivo. Conversely, our data suggest that nesprin 1α2 is the critical nesprin 1 isoform in skeletal muscle and is essential for postnatal viability. Furthermore, the interaction between nesprin 1α2 and kinesin 1 likely plays an important role to correctly position nuclei in developing muscle fibers.

## Materials and methods

### Gene targeting and generation of nesprin 1 mutant mice

Genomic DNA was isolated from R1 embryonic stem (ES) cells and was used to generate nesprin 1α2 and nesprin 1ΔCH targeting constructs as described in our previous study (Zhang et al., 2015). In brief, the construct of nesprin 1ΔCH was generated in the pBluescript II KS^+^ vector in three sections (Fig. 1). The 5′ arm of homology consisted of a 3.48-kb KpnI–SalI fragment that was generated using the primers forward, 5′-GTGGTACCAGATAGAAGAGTTGACCAGCAACC-3′; and reverse, 5′-GCGTCGACACTTCTGACAGACTGACTTGG-3′. The middle fragment containing the first LoxP site, neomycin, flanked by the flippase recombination target sites exon 7F and the second LoxP site was generated using the primers forward, 5′-GCGGATCCGACAAGAGAATTGGCAGGTCCAAAC-3′; and reverse, 5′-GCGGATCCAATGGCAAAGAGGTGTTTGGAG-3′. The 3′ arm consisted of a 5.1-kb Acc65I–NotI fragment that was generated using the primers forward, 5′-GTCCCGGGCTTGTGAACAGAACTTAAAATATCC-3′; and reverse,5′-GAGCGGCCGCATTTGATCATGTCTCCTGGGCCC-3′.

The construct of nesprin 1α2 was generated in the pBluescript II KS^+^ vector in three sections (Fig. 3). The 5′ arm of homology consisted of a 3.74-kb NotI–Acc65I fragment that was generated using the primers forward, 5′-GTGCGGCCGCGTGCCTCTTAAACCTGGCATTAGCG-3′; and reverse, 5′-GACCCGGGTTACGGCTCAAAAGAAAGGGTTCCTG-3′. The middle fragment containing the first LoxP site, neomycin, flanked by the flippase recombination target sites exon 1α2 and the second LoxP site was generated using the primers forward, 5′-CAGGATCCTGCTCTTGCTGGCAGATTACCCTTCTTACC-3′; and reverse, 5′-CTGGATCCCACTTTCAGTTTAGTCTGAAGCCACCC-3′. The 3′ arm consisted of a 3.5-kb SalI–KpnI fragment that was generated using the primers forward, 5′-GTGTCGACCGGAGCCTGTTTACAACTTTGC-3′; and reverse, 5′-GAGGTACCGTTCCTGTCCCTGTCTAGGGTCTGGCC-3′. Both targeting constructs were verified by sequencing and linearized with NotI before electroporation into R1 ES cells at the Transgenic Core Facility at the University of California, San Diego. 600 G418-resistant ES clones for nesprin 1ΔCH and 300 G418-resistant ES clones for nesprin 1α2 were screened for homologous recombination by Southern blotting as described in the next section.

### Southern blot analysis

Genomic DNA was extracted from G418-resistant ES cell clones and mouse tails as previously described (Zhang et al., 2015). ES cell DNA was digested with BamHI and analyzed by Southern blot analysis. A 300-bp fragment for nesprin 1α2 and a 212-bp fragment for nesprin 1ΔCH was generated by PCR using mouse genomic DNA and specific nesprin 1α2 primers (forward, 5′-CCTGAGATACTCTCTGCTGTCTAAC-3′; and reverse, 5′-TTTCAGCTATGAAGACTTTATACAG-3′) and nesprin 1ΔCH primers (forward, 5′-GGTAGAACCATGCTTTCTAGAAC-3′; and reverse, 5′-CATCAAAACCTAGAGACCTGAGC-3′). The PCR product was subsequently radiolabeled using α-[^32^P]dCTP by random priming (Invitrogen). DNA blots were hybridized with the radiolabeled probe and visualized by autoradiography. The WT allele of nesprin 1α2 is represented by a band of 9.2 kb, whereas a band of 7.0 kb represents the correctly targeted mutant allele. The WT allele of nesprin 1ΔCH is represented by a band of 18 kb, whereas a band of 6.0 kb represents the correctly targeted mutant allele.

### Generation and genotyping of mice

Two independent homologous recombinant ES clones for nesprin 1α2 and one homologous recombinant ES clone were microinjected into blastocysts from C57/B6 mice at the Transgenic Core Facility at the University of California, San Diego. Male chimeras were bred with *Sox2*-Cre–expressing female Black Swiss mice to generate germ line–transmitted heterozygous mice (nesprin 1α2^+/−^ and nesprin 1ΔCH^+/−^; Hayashi et al., 2002). Nesprin 1α2^+/−^ and nesprin 1ΔCH^+/−^ mice were subsequently intercrossed to generate homozygous null mutant mice (nesprin 1α2^−/−^ and nesprin 1ΔCH^−/−^). Offspring were genotyped by PCR analysis as described in our previous study (Lin et al., 2016) with mouse tail DNA, and we used the genotyping primers nesprin 1α2 forward, 5′-GAAAATAGCTCATGGTAATATTCACCTCC-3′; and reverse, 5′-GAAATATGAATTTAGAGCCATCAACAGG-3′; and nesprin 1ΔCH forward, 5′-CATTTCATGAATTTTGAGATCCCATTAAG-3′; and reverse 5′-AACTCTGATGAGGCCTCAGAGCTACATG-3′.

### Single fiber isolation for nuclear distance measurements and LINC complex immunofluorescence

To obtain fine single myofibers, TA muscles were isolated from E18.5 embryos and fixed with 4% PFA for >2 d. Fixed muscles were divided into several bundles by pulling the tendon with tweezers, and nonmuscle tissue was removed to the greatest degree possible under a dissection microscope. For nuclear distance measurements, the muscle bundles were macerated in 40% NaOH solution for 30 min at RT and then shaken for 5–10 min to separate the bundle into single myofibers. Isolated myofibers were rinsed twice with PBS, pH 7.3, for neutralization. They were then stained with DAPI and mounted. For immunofluorescence staining, single fibers were permeabilized with PBS containing 0.2% Triton X-100 and then stained with the various antibodies diluted in PBS containing 3% BSA and 0.1% Triton X-100. Antibodies are listed in Table 1. Images were acquired at RT on an IX70 microscope (Olympus) controlled by an RT Deconvolution System (DeltaVision) using oil-immersed 40× 1.30 NA or 100× 1.40 NA objectives with the respective immersion oils (DeltaVision) and a charge-coupled device camera (CoolSNAP HQ; Photometrics) as described previously (Stroud et al., 2011, 2014b). Images were deconvolved using SoftWoRx software, levels were adjusted with ImageJ (National Institutes of Health), and figures were assembled in Photoshop and Illustrator (CS5.1; Adobe).

**Table 1.** List of antibodies used for immunofluorescence staining

**Antibody**	**Source**	**Catalog number**
Nesprin 1	Glenn E. Morris	MANNES1E
Sun1	EMD Millipore	ABT285
Sun1	Abcam	ab103021
Sun2	Epitomics	EPR6557
Emerin	Santa Cruz Biotechnology, Inc.	SC15378
Lamin A/C	Larry Gerace	n/a
Lamin B1	Larry Gerace	n/a
Lamin B2	Larry Gerace	n/a
KLC1/2	Santa Cruz Biotechnology, Inc.	SC25735
KIF5B	Abcam	ab167429
GAPDH	Santa Cruz Biotechnology, Inc.	SC32233
α-actinin	Sigma-Aldrich	A7811
α Tubulin	Sigma-Aldrich	Clone DM1A (T9026)
p150 (glued)	BD	610473

### Nuclear distance measurements

Nuclear lengths and internuclear distances was measured using fibers isolated as in the previous section with ImageJ. In brief, for internuclear distances, a line was drawn between the nuclear centroid to centroid; for nuclear lengths, a line was drawn along the axis of the myofiber between the shortest widths of the nuclei.

### Western blotting

Western blot analysis was performed as described previously (Fang et al., 2016). In brief, protein lysates were run on 4–12% SDS-PAGE gels (Thermo Fisher Scientific) and transferred overnight at 4°C on to polyvinylidene difluoride membrane (Bio-Rad Laboratories). After this, they were blocked for 1 h in wash buffer (TBS with 0.1% Tween-20; TBST) containing 5% milk and incubated overnight at 4°C with the indicated primary antibodies (Table S1) in wash buffer supplemented with 2% milk. Blots were washed and incubated with HRP-conjugated secondary antibody generated in rabbit (1:5,000) or mouse (1:2,000; Dako) for 1 h at RT. Blots were visualized using ECL Chemiluminescence (Bio-Rad Laboratories).

### Antibodies

Antibodies used for immunofluorescence and Western blotting are listed in Table S1.

### Real-time PCR

Total RNA was extracted from mouse TA muscle using TRIzol reagent according to manufacturer’s instructions (Thermo Fisher Scientific). cDNA was synthesized using SuperScript 3 (Thermo Fisher Scientific). RT-PCR reactions were performed using PerfeCTa SYBR green FastMix master mix (Quantabio) in 96-well low-profile PCR plates in a CFX96 Thermocycler (Bio-Rad Laboratories). Primers used are listed in Table S2.

### Statistics

Data are presented as means ± SEM unless indicated otherwise. We used two-tailed Student’s *t* tests or analyses of variance for comparisons among groups as indicated. Analysis was performed using Excel software (Microsoft). P-values of <0.05 were considered significant.

### Animal procedures and study approval

All animal procedures were approved by the University of California, San Diego, Animal Care and Use Committee. The University of California, San Diego, has an Animal Welfare Assurance (A3033-01) on file with the Office of Laboratory Animal Welfare and is fully accredited by AAALAC International.

### Online supplemental material

Fig. S1 shows levels of LINC proteins and nuclear lamina proteins in nesprin 1 mutant mice. Fig. S2 shows levels of nesprin 2 short and long isoforms in nesprin 1 mouse models. Fig. S3 displays microtubule organization in TA muscles isolated from nesprin 1ΔCH and 1α2 mice. Video 1 shows a 3D stack of a nesprin 1α2 KO muscle fiber at 40×. Video 2 shows a 3D stack of nesprin 1α2 KO muscle fiber at 100×. Video 3 shows a 3D stack of WT muscle fibers at 100×. Table S1 shows the primer sequences used for RT-PCR. Table S2 shows the antibodies used in the study.

## Supplementary Material

Supplemental Materials (PDF)

Video 1

Video 2

Video 3
