# Supplementary material for: Nesprin 1α2 is essential for mouse postnatal viability and nuclear positioning in skeletal muscle
Source: J Cell Biol. 2017 Jul 3;216(7):1915–24. doi: 10.1083/jcb.201612128 (PMC5496623; doi:10.1083/jcb.201612128)
Supplement: Supplemental Materials (PDF) [file JCB_201612128_sm.pdf]

Stroud et al., https://doi.org/10.1083/jcb.201612128

THE JOURNAL OF CELL BIOLOGY

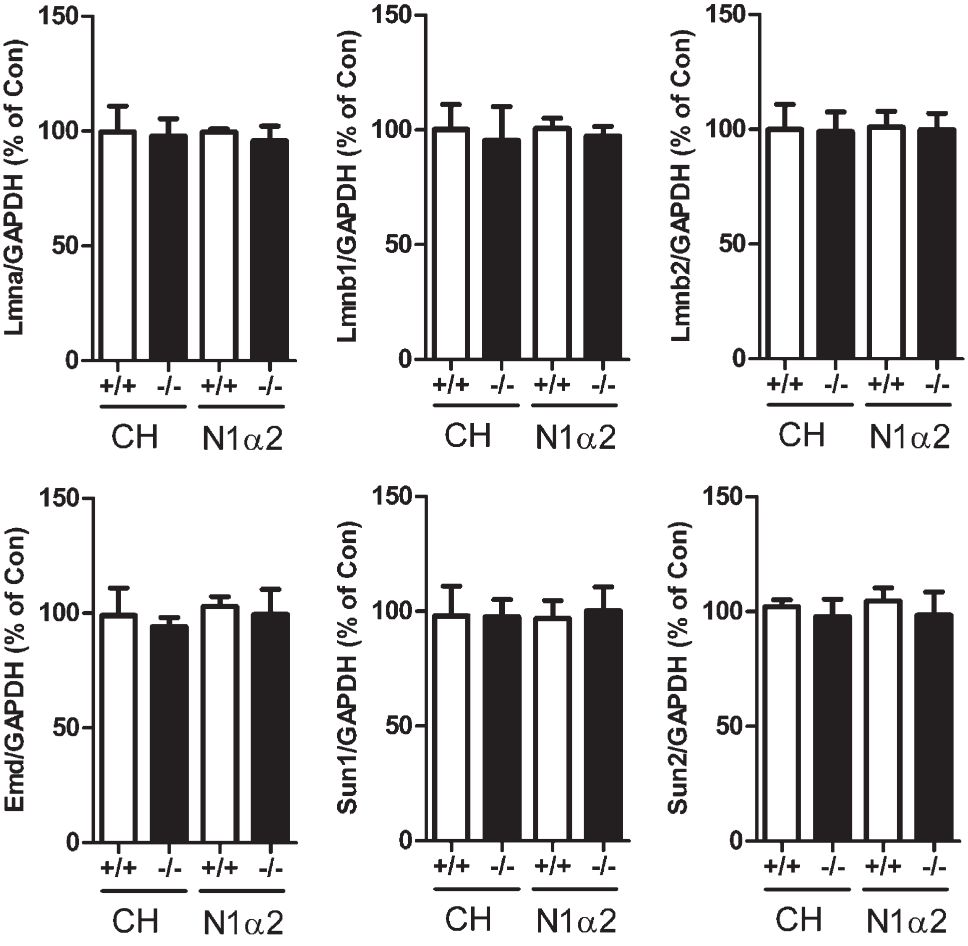

Figure S1. **Levels of LINC proteins and nuclear lamina proteins.** qRT-PCR was performed on mRNA isolated from TA skeletal muscle of nesprin 1ΔCH (CH), nesprin 1α2 KO (N1α2), and their respective WT littermates. Note that levels of the NE components were unchanged in both nesprin mutants. Con, control; Emd, emerin; Lmna, Lamin A; Lmnb1, Lamin B1; Lmnb2, Lamin B2. *n* = 3 per genotype.

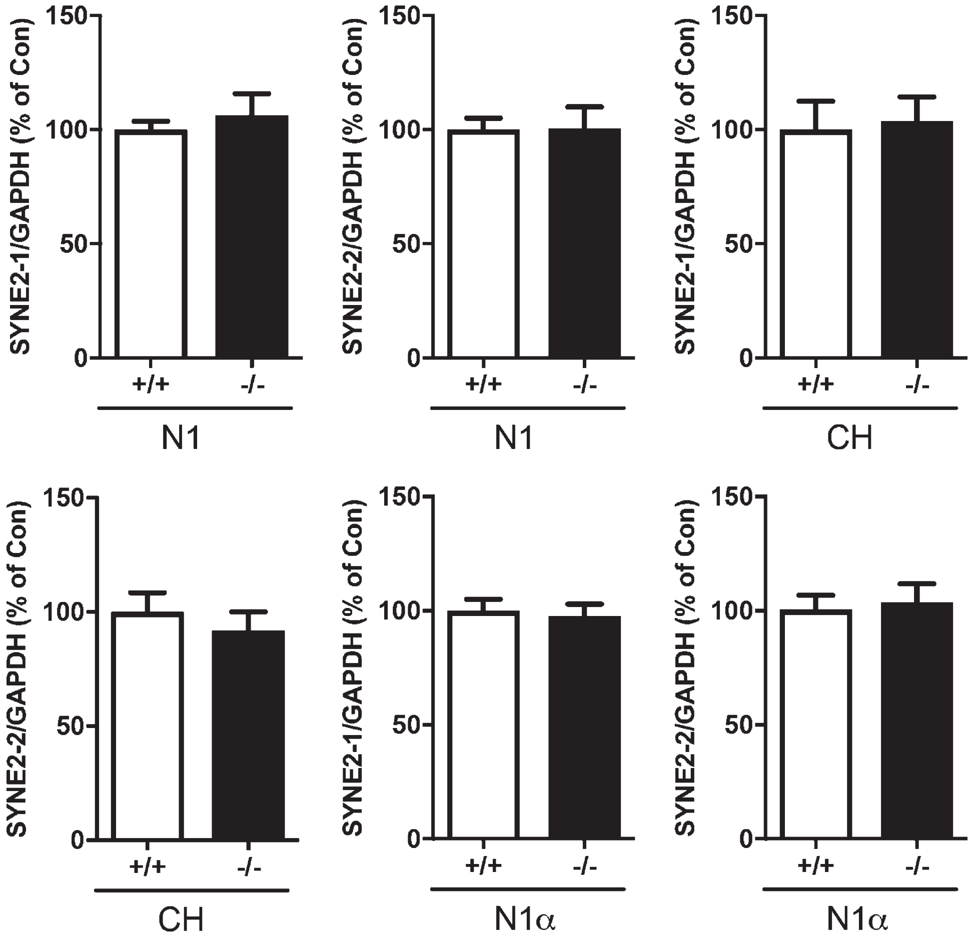

Figure S2. **Levels of nesprin 2 short and long isoforms in nesprin 1 mouse models.** qRT-PCR was performed on mRNA isolated from the TA skeletal muscle of nesprin 1 GKO (N1), nesprin 1ΔCH (CH), nesprin 1α2 KO (N1α2), and their respective WT littermates. Note that the levels of nesprin 2 short (Syne2-1) and long (Syne2-2) isoforms were unchanged in all three mice. *n* = 3 per genotype. Con, control.

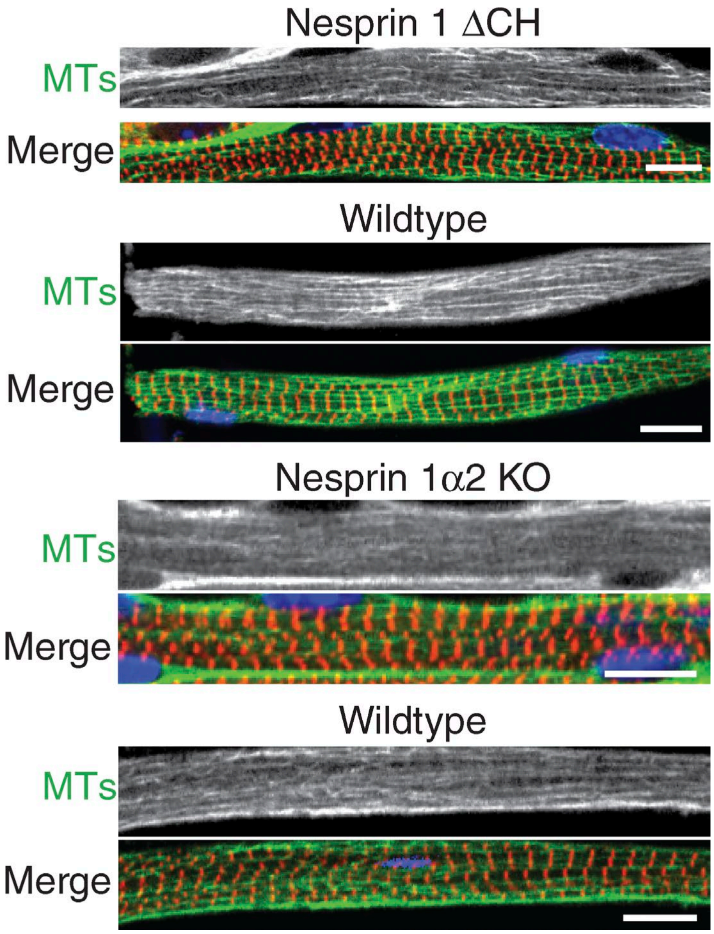

Figure S3. **Microtubule organization is unaffected in TA muscles isolated from nesprin 1ΔCH and 1α2 mice.** TA muscles isolated from the indicated genotypes were stained for α tubulin to mark microtubules (MTs; green). Merge images represent α-actinin (red), DAPI (blue), and MTs (green). Note that MT organization is similar between nesprin 1 mutants and WTs. Bars, 10 mm.

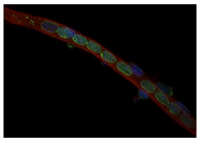

Video 1. **3D stack of nesprin 1α2 KO muscle fiber.** Individual muscle fiber was stained using antibodies directed against Lamin A/C (green), α-actinin (red), and DAPI (blue). Z series images using a 40× objective were taken through the fiber at 0.2-µm intervals, deconvolved, and reconstructed using the McMaster Biophotonics Facility plugin "Stacks Z functions." X and Y rotation with parameters set to 360° rotation in ImageJ. Playback is at 14 frames per second.

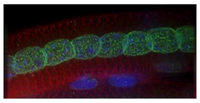

Video 2. **3D stack of nesprin 1α2 KO muscle fiber.** Individual muscle fiber was stained using antibodies directed against Lamin A/C (green), α-actinin (red), and DAPI (blue). Z series images using a 100× objective were taken through the fiber at 0.2-µm intervals, deconvolved, and reconstructed using the McMaster Biophotonics Facility plugin "Stacks Z functions." X and Y rotation with parameters set to 360° rotation in ImageJ. Playback is at 14 frames per second.

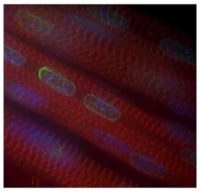

Video 3. **3D stack of WT muscle fibers.** Individual muscle fiber was stained using antibodies directed against Lamin A/C (green), α-actinin (red), and DAPI (blue). Z series images using a 100× objective were taken through the fiber at 0.2-µm intervals, deconvolved, and reconstructed using the McMaster Biophotonics Facility plugin "Stacks Z functions." X and Y rotation with parameters set to 360° rotation in ImageJ. Playback is at 14 frames per second.

Table S1. **Primer sequences used for qRT-PCR**

Primer	Forward (5′–3′)	Reverse (5′–3′)
Emd, qRT-PCR	TTGGGCCCTGTCTGTAGG	TAGGAGAATGAAGAAGATGACGAGT
Sun1, qRT-PCR	ATGGAACACATTCCAAAGACACTAT	GAGCTCTACTATCTGGAAGGCTTG
Sun2, qRT-PCR	CACTCGCTACTCTCAGGATGATAA	TAGGACTCTCGAACCACAGACTC
Lamin A, qRT-PCR	CCTTCGCATCACTGAGTCTG	AGTCCCCCTCCTTCTTGGT
Lamin B1, qRT-PCR	AGATCGAGCTGGGCAAGTT	GCTGGGCAATCTGATCTTTC
Lamin B2, qRT-PCR	AGAAGGAGGAGCTCCGTGA	TGCACCTTTCCAATTTCAATC
Nesprin 1 exons 8F–9F qRT-PCR	GCTGCAGTCTCTATCCAGCAGT	GATGCCTAGCTGTGTTTCAGCG
Nesprin 2 exons 86–88 (long isoforms) qRT-PCR	CCTTGAGGCCCTTCAGGATG	TCTGCGAGGTCTTGGTTCAC
Nesprin 2 exons 110–112 (short isoforms) qRT-PCR	AGTGCCAGGACTTTCACCAG	CTTCACCAGAAGGCTGGAGG

Table S2. **Primers used for semiquantitative PCR**

Primer	Sequence (5′–3′)
9B	CACTGGAGTCACAGATGAGGC
9B′	CACACACTTTCTGCTTTGCCATAG
7B	GCTGCCACTTCTGAAGGTTCTG
18B	CATCTCCTGGACCTCATGGC
15B	GCCACAGTCGCCACGTCTCT
E1A2	GGACTGAGCCTTTCGCTCTG
GAPDH forward	CACAGTCAAGGCCGAGAATGGGAA
GAPDH reverse	GTGGTTCACACCCATCACAAACATG
